# The improve immune function of haemofiltration combine with HA haemoadsorption in HLA - DR low expression of sepsis patients

**DOI:** 10.1186/2197-425X-3-S1-A418

**Published:** 2015-10-01

**Authors:** L Ying

**Affiliations:** Zhejiang University, Shaoxing, China

## Objectives

To investigate that the treatment of the hemofiltration combine with HA hemoadsorption to improve the immune function in HLA-DR low expression of sepsis patients.

## Methods

60 sepsis patients with HLA -DR expression < 30% of patients was selected, and divided them randomly into experimental group and control group, each group has 30 cases, and they were treated with sepsis standard operating procedures, on the basis of that, the experimental group increases the treatment of hemofiltration combine with the HA hemoadsorption in 1-3 days, the CVVH mode, and the former dilution volume is 4L/h, the hemofilter HF2000 series blood adsorber HA -330H. On the 0, 3, 5, 7 days of treatment, detecting the two groups of patients' expression level of the peripheral blood mononuclear cells of HLA - DR; and evaluate APACHE II scores of two groups of patients, duration of mechanical ventilation, ICU stay and the differences of 28 days survival rate between the two groups.

## Results

Patients' HLA-DR expression of two groups was all < 30% before treatment, and there is no statistical difference, (25.9 ± 7.3% vs 26.4 ± 6.7%, p > 0.05). In the treatment of 3, 5, 7 days ,the HLA - DR expression of the experimental group increased significantly, and apparently higher than the control group, there are statistical differences, including after the treatment 3 days that HLA - DR (38.9 ± 8.6% vs 29.3 ± 7.1%, P < 0.05), 5 days' (42.7 ± 9.2% vs 31.4 ± 6.5%, P < 0.05), 7 days' (40.9 ± 8.5% vs 29.4 ± 6.7%,

P < 0.05). APACHE II grade shows that there is no significant difference between the two groups before treatment (22.4 ± 5.3 vs 21.7 ± 6.2, P > 0.05). After 3,5, and 7 days' treatment, we can see the APACHE II grade of the experimental group decreased, which is obviously less than the control group, and there is statistical difference between two groups. The APACHE II grade after 3 days treatment is (18.6 ± 3.6 vs 20.5 ± 4.3, p < 0.05), 5 days' treatment is (15.8 ± 3.9 vs 21.1 ± 4.4, p < 0.05) and 7 day' treatment is (14.9 ± 4.2 vs 19.8 ± 3.7, p < 0.05). Comparing the experimental group to the control group, there is a statistical difference in the duration of mechanical ventilation (13.3 ± 3.4 vs 18.9 ± 4.5, P < 0.05), ICU stay (20.7 ± 3.9 vs 26.8 ± 4.7, P < 0.05) and the 28 days survival rate (83.3% vs 73.3%, P < 0.05).

## Conclusions

Hemofiltration combined with HA hemoadsorption can improve the expression level of HLA-DR low expression of sepsis patients, to some degree ,improve immune function of sepsis patients and prognosis.

